# ^68^Ga-DOTA chelate, a novel imaging agent for assessment of myocardial perfusion and infarction detection in a rodent model

**DOI:** 10.1007/s12350-019-01752-6

**Published:** 2019-05-29

**Authors:** Anu Autio, Sauli Uotila, Max Kiugel, Ville Kytö, Heidi Liljenbäck, Nobuyuki Kudomi, Vesa Oikonen, Olli Metsälä, Semi Helin, Juhani Knuuti, Antti Saraste, Anne Roivainen

**Affiliations:** 1 Turku PET Centre, University of Turku, Kiinamyllynkatu 4-8, 20521 Turku, Finland; 2 MediCity Research Laboratory, University of Turku, Turku, Finland; 3 Heart Center, Turku University Hospital and University of Turku, Turku, Finland; 4 Turku Center for Disease Modeling, University of Turku, Turku, Finland; 5 Department of Medical Physics, Faculty of Medicine, Kagawa University, Kagawa, Japan; 6 Turku PET Centre, Turku University Hospital, Turku, Finland

**Keywords:** PET, myocardial perfusion, myocardial infarction, rat

## Abstract

**Background:**

Magnetic resonance imaging (MRI) with Gadolinium 1,4,7,10-tetraazacyclododecane-*N*′,*N*″,*N*′′′,*N*″″-tetraacetic acid (Gd-DOTA) enables assessment of myocardial perfusion during first-pass of the contrast agent, while increased retention can signify areas of myocardial infarction (MI). We studied whether Gallium-68-labeled analog, ^68^Ga-DOTA, can be used to assess myocardial perfusion on positron emission tomography/computed tomography (PET/CT) in rats, comparing it with ^11^C-acetate.

**Methods:**

Rats were studied with ^11^C-acetate and ^68^Ga-DOTA at 24 hours after permanent ligation of the left coronary artery or sham operation. One-tissue compartmental models were used to estimate myocardial perfusion in normal and infarcted myocardium. After the PET scan, hearts were sectioned for autoradiographic detection of ^68^Ga-DOTA distribution.

**Results:**

^11^C-acetate PET showed perfusion defects and histology showed myocardial necrosis in all animals after coronary ligation. Kinetic modeling of ^68^Ga-DOTA showed significantly higher *k*_1_ values in normal myocardium than in infarcted areas. There was a significant correlation (*r *= 0.82, *P *= 0.001) between *k*_1_ values obtained with ^68^Ga-DOTA and ^11^C-acetate. After 10 minutes of tracer distribution, the ^68^Ga-DOTA concentration was significantly higher in the infarcted than normal myocardium on PET imaging and autoradiography.

**Conclusions:**

Our results indicate that acute MI can be detected as reduced perfusion, as well as increased late retention of ^68^Ga-DOTA.

**Electronic supplementary material:**

The online version of this article (10.1007/s12350-019-01752-6) contains supplementary material, which is available to authorized users.

## Introduction

Gadolinium (Gd)-labeled chelates are widely used magnetic resonance imaging (MRI) contrast agents. Cardiac MRI with Gd chelates enables assessment of myocardial perfusion during the first-pass of the contrast agent and detection of myocardial infarction (MI) using the delayed-enhancement technique.^1^^–^6 Delayed-enhancement imaging is based on the accumulation and retention of contrast agent in the infarcted myocardium due to the loss of cell membrane integrity, and hence reflects increased extracellular tissue volume.^7^

Positron emission tomography/computed tomography (PET/CT) perfusion imaging enables quantification of myocardial blood flow (MBF) and myocardial flow reserve (MFR). As it does not require an on-site cyclotron, rubidium-82 (^82^Rb) chloride is the most widely used tracer for assessment of myocardial perfusion with PET. Quantification of myocardial perfusion on PET with the cyclotron products nitrogen-13-labeled ammonia (^13^N-ammonia) and oxygen-15-labeled water (^15^O-water) has been well validated.^8^,^9^ Another validated tracer for myocardial perfusion imaging is the ^11^C-acetate used as a control in this study.^10^

Gallium-68-labeled 1,4,7,10-tetraazacyclododecane-*N*′,*N*″,*N*′′′,*N*″″-tetraacetic acid (^68^Ga-DOTA) chelate has similar kinetics to the Gd-DOTA used in MRI,^11^ and is feasible for imaging of blood flow.^12^,^13^ Our research group found evidence that imaging of increased accumulation of ^68^Ga-DOTA PET may be useful for detecting conditions associated with increased blood flow and vascular permeability, such as adenosine-induced increase in myocardial perfusion and inflammatory lesions.^12^ This novel approach to PET perfusion imaging is generator-based and provides an opportunity for imaging centers distant to a radiochemistry unit to detect and quantify blood flow. ^68^Ga is a positron-emitting radionuclide that is readily available from a ^68^Germanium (^68^Ge)/^68^Ga generator system possessing a 1 year life span (^68^Ge *T*_1/2_ = 270.8 days). In contrast to most positron-emitting radionuclides that require a cyclotron for production, the ^68^Ge/^68^Ga generator provides low-cost and easy access to a positron-emitting radionuclide. However, it is not known whether ^68^Ga-DOTA PET can be used to detect abnormal myocardial perfusion and cell necrosis associated with MI.

In this study, we aimed to validate ^68^Ga-DOTA PET imaging measurement of myocardial perfusion, comparing it with ^11^C-acetate, and comparing the kinetics of ^68^Ga-DOTA between normal and infarcted myocardium in rats. Translation to a clinical setting should be feasible for ^68^Ga-DOTA, as Gd-DOTA is already in clinical use (trade names Artirem®, Dotarem®).^14^

## METHODS

### Animal Model

The study protocol was approved by the national Animal Experiment Board in Finland and the Regional State Administrative Agency for Southern Finland, and carried out in compliance with the European Union directives relating to the conduct of animal experimentation. In total, 12 male Sprague-Dawley rats were used. MI was induced by permanent ligation of the left coronary artery (LCA), as previously described.^15^^–^18 Briefly, 0.2 mg·kg^−1^ buprenorphine (Temgesic; Schering-Plough, Espoo, Finland) was administered intramuscularly for analgesia prior to operation. Anesthesia was induced by a combination of inhaled isoflurane and subcutaneous injection of 10 mg·kg^−1^ xylazine (Rompun; Orion Pharma, Espoo, Finland) and 90 mg·kg^−1^ ketamine (Ketaminol; Orion Pharma). Body temperature was maintained using a heating pad. The rats were intubated and connected to a rodent ventilator (TOPO dual mode ventilator; Kent Scientific, Torrington, CT, USA).^19^

The heart was exposed by a left lateral thoracotomy of the fourth intercostal space, the pericardium was opened, and the LCA was ligated near to its origin. Ligation was confirmed visually as a pale appearance of the myocardium at risk. The ribs, muscle layer, and skin were tightly sewn with a dissolving string. The anesthesia was reversed after the operation with an intramuscular injection of 1 mg·kg^−1^ atipamezole (Antisedan; Orion Pharma). The sham operation consisted of all the same procedures except that the suture was not tightened around the LCA. Operative mortality in both coronary ligation and sham groups was ~ 25%, as previously described.^15^

### Radiochemistry

^68^Ga-DOTA synthesis was performed as previously described.^12^ Briefly, ^68^Ga was obtained from a ^68^Ge/^68^Ga generator (Eckert&Ziegler, Valencia, CA, USA) by elution with 0.1 M HCl. DOTA chelate (35 nmol in aqua; Macrocyclics, Dallas, TX, USA) was added, and the mixture was incubated at + 100°C for 15 minutes. Radiochemical purity of the final product was analyzed by high-performance liquid chromatography (HPLC) using μBondapak column and trifluoroacetic acid/acetonitrile/H_3_PO_4_ gradient. Retention time for ^68^Ga-DOTA was 3.9 ± 0.1 minutes and for unbound ^68^Ga 14.6 ± 0.2 minutes.

^11^C-Acetate was synthesized as previously described.^20^ For quality assessment, the final product was analyzed by HPLC.

### PET/CT

The rats that survived surgery were imaged using small-animal PET/CT (Inveon Multimodality; Siemens Medical Solutions, Knoxville, TN, USA) at 24 ± 4 hours (*n *= 6) after coronary ligation or at 24 ± 4 hours (*n *= 6) after the sham operation. The rats were anesthetized using 1.5% isoflurane, and their body temperature was maintained with a heating pad throughout the imaging. First, a 10-minutes ^11^C-acetate PET (27 ± 12 MBq) was performed to evaluate myocardial perfusion and to localize the infarcted area. Then, after five half-lives of ^11^C, ^68^Ga-DOTA imaging was performed. To evaluate the myocardial kinetics of the tracer, the rats in the ligation group were injected with a bolus of 29 ± 3 MBq of ^68^Ga-DOTA and PET imaged for 30 minutes. Both ^11^C-acetate and ^68^Ga-DOTA PET acquisitions were started prior to injection to catch the first-pass peak of the imaging tracer. Images were collected according to the following time frames: 30 × 3 seconds, 9 × 10 seconds, 4 × 30 seconds, 5 × 60 seconds, and 4 × 300 seconds. Immediately after PET, 200 μL of intravascular iodinated eXIATM160XL contrast agent (Binitio Biomedical Inc, Ottawa, ON, Canada) was injected and high-resolution CT was acquired to delineate the myocardial borders. Sham-operated rats were injected with 14 ± 1 MBq of ^68^Ga-DOTA and PET/CT imaged as described above.

Alignment of PET and CT images was automatically performed and confirmed visually according to anatomical landmarks. Image analysis was performed using Carimas v.2.8 software (Turku PET Centre, Turku, Finland). Regions of interest (ROIs) were defined on the high-resolution CT image to delineate the infarcted myocardium (verified by ^11^C-acetate perfusion images) and remote myocardium. Results are reported as standardized uptake values (SUVs).

The kinetic parameters for both ^68^Ga-DOTA and ^11^C-acetate were determined using the Carimas 2.8 heart plugin modeling option. For ^68^Ga-DOTA, an input function was obtained from the left ventricle (LV), which was identified using high-resolution contrast-enhanced CT. The uptake rate (*k*_1_) was estimated assuming a one-tissue compartmental model (1TCM), as we previously validated.^12^ Modeling for ^11^C-acetate was performed using a tracer-specific mode readily available in the Carimas software.^15^ An ^11^C-acetate *k*_1_ polar map was used to determine specific segments in the normal areas in the septum and infarcted area in the anterior or anterolateral LV wall, with these same segments being used to compare the ^68^Ga-DOTA kinetics. In addition to perfusion, the myocardial infarct area was determined as the percent of non-viable tissue according to the *k*_1_ polar maps. The polar maps were normalized to the highest *k*_1_ value in the viable part of the myocardium, which was set as the 100% *k*_1_ value, and a cutoff of < 60% of the *k*_1_ value was used to determine the infarcted area.^21^

### Autoradiography

A subgroup of MI rats (*n *= 3) were further analyzed to determine the microscopic distribution of the ^68^Ga-DOTA at 30 minutes post-injection of the tracer. The LV was frozen in cooled isopentane and sliced into serial transverse cryosections of 8 and 20 μm at 1 mm intervals (five to six intervals per heart) from apex to base. Distribution of ^68^Ga-DOTA in the infarcted and remote myocardium was analyzed by autoradiography of tissue sections. Air-dried sections were apposed to an imaging plate (BAS-TR2025; Fuji Photo Film Co. Tokyo, Japan), and after more than two radionuclide half-lives, the plates were scanned with a Fuji BAS-5000 analyzer (internal resolution of 25 μm). Tracer accumulation was measured as counts per area (photostimulated luminescence per square millimeter, PSL/mm^2^) with TINA software v.2.1 (Raytest Isotopenmessgeräte, GmbH, Straubenhardt, Germany).

After co-registration of autoradiography and histological images, ROIs were defined in the infarcted and remote areas of the LV. The background area count densities were subtracted from the image data. After autoradiography, the serial LV cryosections were stained with hematoxylin-eosin (HE) for histology.

### Statistical Analysis

All results are expressed as mean ± standard deviation (SD). Correlation coefficients were computed using linear regression analysis. Student’s *t*-test was used for comparison between two groups. A *P* value less than 0.05 was considered statistically significant. All statistical analyses were conducted using Origin 7.5 software (Microcal Software, Inc., Northampton, MA, USA).

## RESULTS

### Radiochemistry

The radiochemical purity of ^68^Ga-DOTA was 99% ± 0.3% (*n *= 4), and the molar activity was 9.1 ± 3.5 MBq·nmol^−1^ (*n *= 4). The radiochemical purity of ^11^C-acetate was consistently > 99%.

### Tracer Kinetics in the Infarcted vs. Normal Myocardium

**Figure 1:**
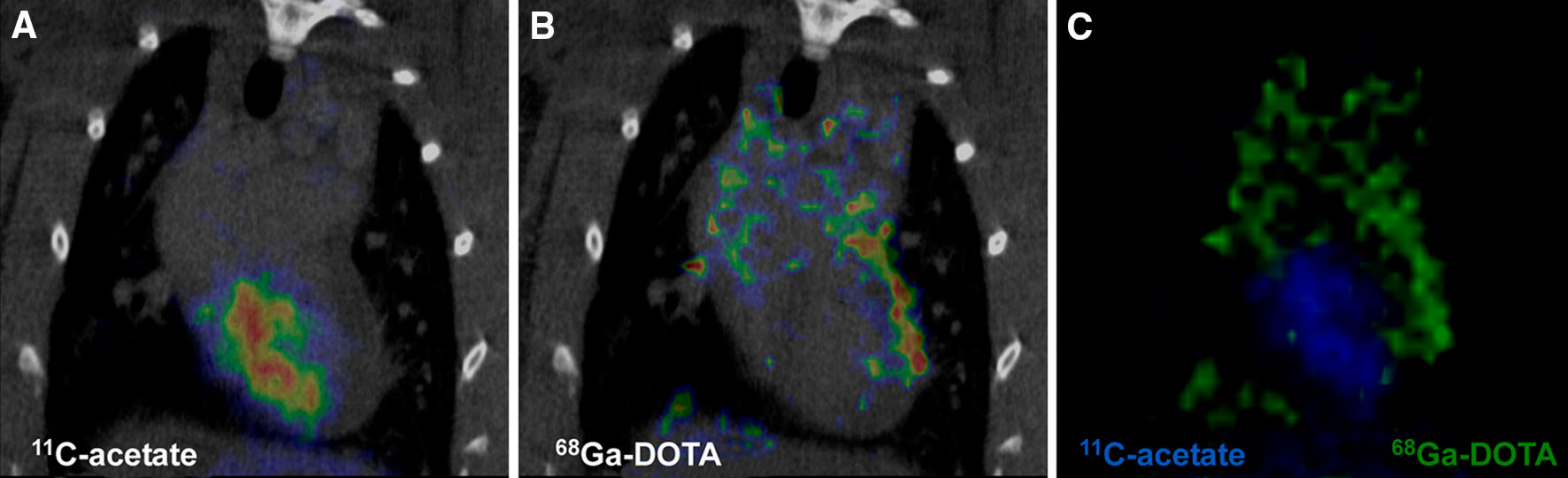
Representative coronal PET/CT images of (**A**) ^11^C-acetate (sum image 30-90 seconds post-injection), (**B**) ^68^Ga-DOTA (sum image 20-30 minutes post-injection), and (**C**) combined ^11^C-acetate (blue) and ^68^Ga-DOTA (green) in a rat with coronary ligation. Note the high ^11^C-acetate uptake in the infero-posterior wall of the LV and defective uptake in the anterior wall subtended by the ligated coronary artery. ^68^Ga-DOTA is present in the defect area at this time point

^68^Ga-DOTA radioactivity concentration declined rapidly after initial peak in the remote and infarcted areas, but some remaining radioactivity was visible in the infarcted area 10 minutes after injection of the ^68^Ga-DOTA tracer. Infarct area was also visualized as a defect area with ^11^C-acetate in every MI animal (Fig. 1A). An example PET/CT image of the remaining radioactivity in the infarcted rat myocardium 30 minutes post-injection of ^68^Ga-DOTA is shown in Figure 1B.

**Figure 2:**
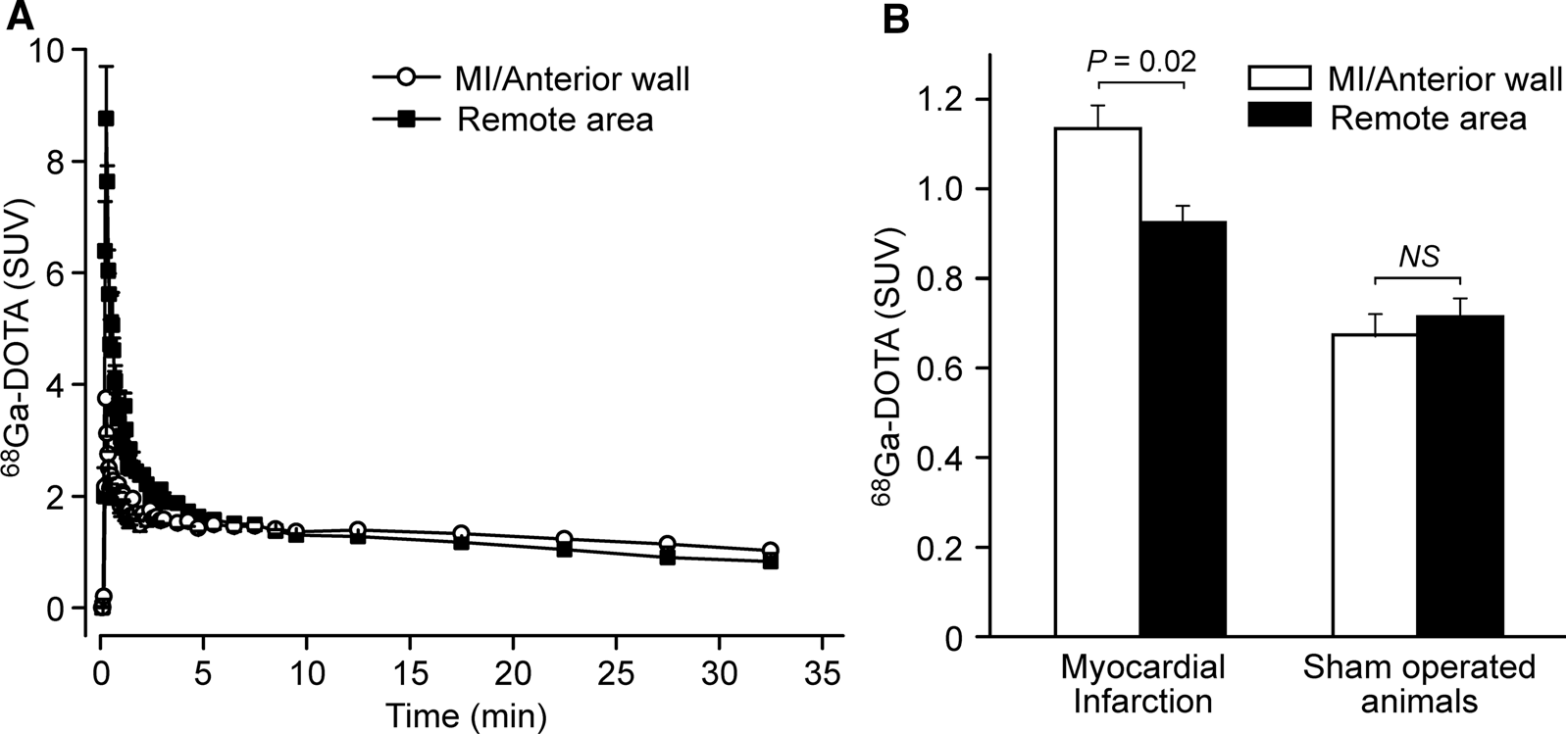
(**A**) Radioactivity concentration as a function of time (time-activity curves) for myocardial ^68^Ga-DOTA uptake in rats with coronary ligation (mean, *n *= 6). Radioactivity peaks were higher in the remote myocardium than in the infarcted area in the initial distribution phase of the ^68^Ga-DOTA. (**B**) Tracer uptake in the heart at the end of the 30 minutes imaging study in MI and sham-operated animals demonstrates late phase contrast enhancement in the infarcted myocardium

A peak was detected in the myocardial uptake of ^68^Ga-DOTA within 20 seconds post-injection (Fig. 2B). At this time point, the peak in the MI rat group was higher in the remote myocardium (SUV: 8.8 ± 0.93) than in the infarcted area (SUV: 3.8 ± 0.67, *P *= 0.0014). At the end of the 30 minutes imaging study (25-30 minutes post-injection), the infarcted area (SUV: 1.13 ± 0.13) showed significantly higher uptake than the remote myocardium (SUV: 0.93 ± 0.087, *P *= 0.023; Fig. 2B). There was no difference between the anterior wall (SUV: 0.67 ± 0.13) and the septum (SUV: 0.72 ± 0.10) in the sham-operated animals (*P *= 0.11). The MI/remote ratio (1.2 ± 0.18) in the MI group was significantly higher than the anterior wall/septum ratio (0.93 ± 0.087) in the sham-operated control group (*P *= 0.0049).

### Determination of MBF

**Figure 3:**
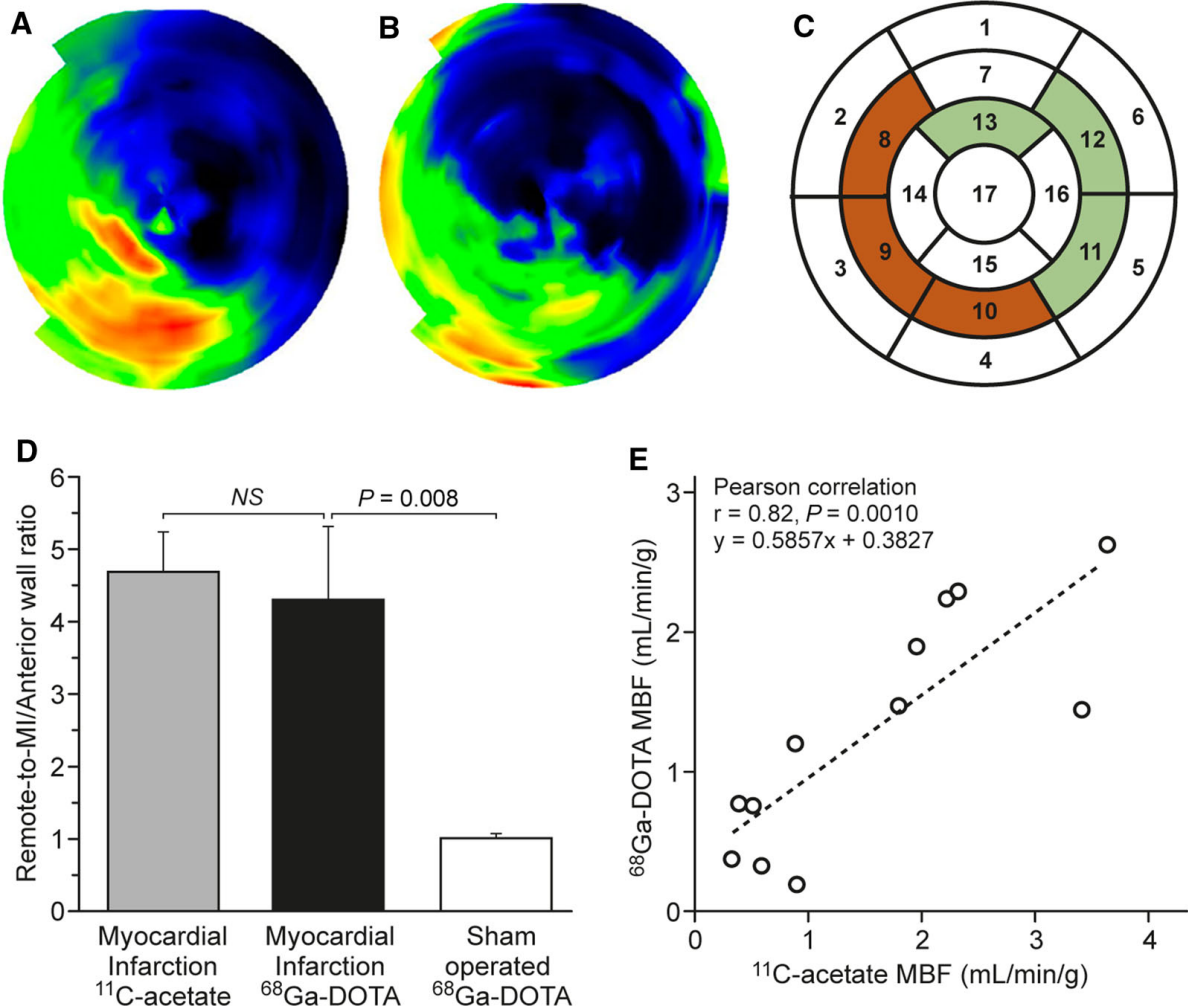
Polar maps of k_1_ values obtained with (**A**) ^11^C-acetate and (**B**) ^68^Ga-DOTA in a rat with coronary ligation. **(C)** Representation of segments used in MBF quantification. Segments marked red were used for quantification of MBF on infarcted area whereas areas in green were used for quantification of remote area. Selected segment varied from animal to animal according localization of infarcted area. Same segment was used for both ^11^C-acetate and ^68^Ga-DOTA MBF quantification per animal. **(D)** Quantification of the remote/MI ratio of *k*_1_ values in MI rats imaged with ^11^C-acetate and ^68^Ga-DOTA, and a sham-operated animal imaged with ^68^Ga-DOTA, reveals comparable kinetics. **(E)** Correlation between ^68^Ga-DOTA and ^11^C-acetate *k*_1_ values is statistically significant. *y* = *ax* + *b* is the linear regression formula used to fit the *k*_1_ data of ^11^C-acetate and ^68^Ga-DOTA

^68^Ga-DOTA k_1_ values reflecting myocardial perfusion were calculated from the PET image data by application of a 1TCM. Polar maps demonstrated higher k_1_ values in the normal myocardium than infarcted myocardium using either ^68^Ga-DOTA or ^11^C-acetate (Fig 3A, 3B). Remote/MI ratios of k_1_ values were 4.7 ± 0.6 (*P *< 0.001) for ^11^C-acetate and 4.3 ± 1.0 (*P *< 0.001) for ^68^Ga-DOTA (Fig. 3D). Regression analysis showed that the ^68^Ga-DOTA values were closely comparable to the ^11^C-acetate values (*y* = 0.59*x* + 0.38, *r *= 0.82, *P *= 0.0010, Fig. 3D). The ratios of the remote to MI k_1_ values showed no significant differences between the ^11^C-acetate and ^68^Ga-DOTA k_1._ In the sham-operated animals, the ratio of k_1_ values for ^68^Ga-DOTA in the corresponding areas in the septum and anterior wall was 1.0 ± 0.06 (*P *= 0.8, Fig. 3d). When the threshold of 60% of maximum flow was used to define viable myocardium, the perfusion defect size was slightly overestimated with ^68^Ga-DOTA (64% ± 3.9%) in comparison with ^11^C-acetate (57% ± 1.8%), but this difference was not significant (*P *= 0.13).

### Autoradiography

**Figure 4:**
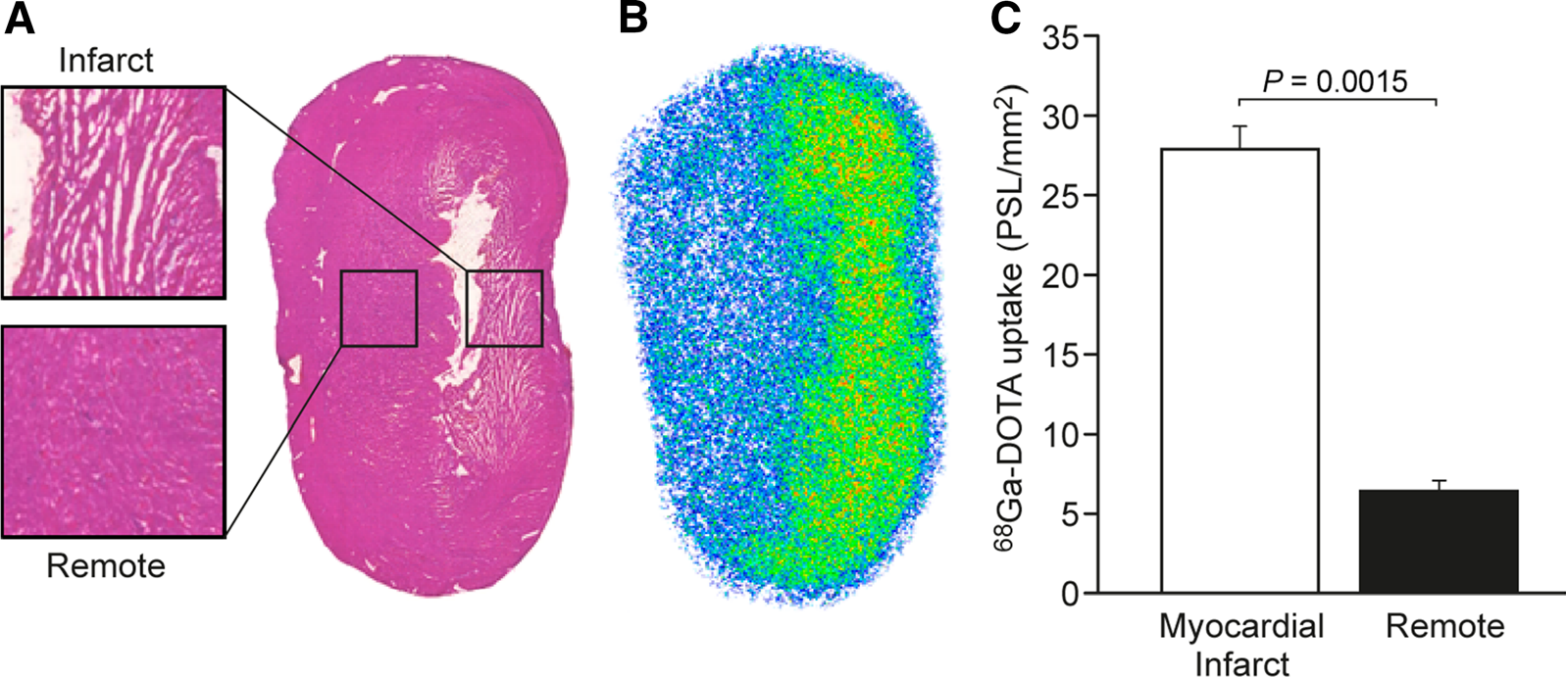
(**A**) Hematoxylin-eosin staining of the left ventricle shows defective myocardium that co-localizes with higher radioactivity concentration. **(B)** Autoradiography of the left ventricle myocardium 30 minutes post-injection of ^68^Ga-DOTA in a rat with myocardial infarction due to coronary ligation shows higher retention of ^68^Ga-DOTA than in remote myocardium. (**C**) Quantification of autoradiography analysis shows significantly higher radioactivity concentration in the ligated area in comparison with remote myocardium

A representative autoradiograph of myocardial ^68^Ga-DOTA uptake at 30 minutes post-injection is shown in Figure 4. Infarcted area, which was confirmed by histology (Fig. 4A), demonstrated a necrotic core surrounded by granulated tissue that was seen in the anterior or anterolateral LV wall in all animals that underwent surgical coronary ligation. There was a local increase in ^68^Ga-DOTA tracer accumulation in the anterolateral wall of the LV, which was co-localized with the histologically infarcted area in the rats with coronary ligation. In the rats with coronary ligation, the average ^68^Ga-DOTA radioactivity concentration in the infarcted area was 4.4-fold higher than in the remote myocardium (28 ± 1.5 vs. 6.5 ± 0.7 PSL·mm^2^, *P = *0.0015).

## DISCUSSION

Gd based MRI is safe, accurate, and practical for the imaging of patients with cardiac events.^5^^–^7 Both first-pass perfusion imaging and delayed contrast enhancement have been utilized for this chelate in cardiac MRI. In the current study, our investigations provide evidence that ^68^Ga-DOTA has similar potential for the measurement of MBF, as well as showing a delayed washout in acutely infarcted myocardium. The ^68^Ga-DOTA works in this setting as a dual-function tracer. In the first-pass and early time frames it can be used to model blood perfusion in the remote myocardium. It is only at later time points when the tracer is accumulated in the defect area due to increased extracellular space. The PET perfusion imaging using tracer kinetic modeling allowed quantitative measurements of absolute MBF. This method is suitable for several applications in research and clinical diagnostics. In rat models, the ^68^Ga-DOTA chelate is comparable to two gold-standards of perfusion imaging, ^15^O-water, as we have previously shown,^12^ and now with ^11^C-acetate. This novel tool would offer the opportunity to in vivo image blood perfusion at imaging sites more distant from cyclotron facility.

Elevated radioactivity concentration was seen in the infarcted areas in the later time points of the ^68^Ga-DOTA imaging, with this probably reflecting the delayed washout kinetics of the tracer distributed in the increased extracellular space, i.e., delayed contrast enhancement. This finding is analogous to the late-enhancement imaging used in contrast-enhanced cardiac MRI, which allows visualization of the infarcted myocardium where cells have lost their membrane integrity or myocardium has been replaced by fibrosis. Higher radioactivity concentration in the infarcted areas was confirmed by autoradiography of the LV tissue sections.

We previously showed the feasibility of ^68^Ga-DOTA chelate for the detection of increased perfusion in inflammation foci,^12^ detecting higher ^68^Ga-DOTA signal at the site of inflamed skin/muscle in comparison with healthy muscle, thereby suggesting that inflammation-induced changes in blood flow and vascular permeability can be detected with ^68^Ga-DOTA. In addition, we were able to detect increased perfusion in healthy rat heart following induction of pharmacological hyperemia caused by intravenous infusion of adenosine. The MFR measured with ^68^Ga-DOTA was comparable to that measured with ^15^O-water, which is a tracer used for PET imaging of MBF.^22^^–^24

The in vivo imaging of MBF is difficult in small animals due to the limited spatial and temporal resolution of PET scanners, partial-volume effects (PVEs), spill over, and the high positron energy of ^68^Ga. However, studies have indicated the possibility of imaging myocardial perfusion in rat heart with several different tracers.^10^,^23^^–^25 Tracers that have been successfully used for the measurement of myocardial infarct size in small animals include 2-deoxy-2-^18^F-fluoro-*D*-glucose (^18^F-FDG), which is a marker of viable myocardium.^25^^–^27 Here, we demonstrated that ^68^Ga-DOTA has potential for the measurement of myocardial perfusion in small animals. The results were validated using ^11^C-acetate, which is well established for the imaging of myocardial perfusion.^10^ The quantitative analysis method of ^11^C-acetate is based on fitting 1TCM parameters *K*_1_, *k*_2_, and *V*_b_ to the tissue curves covering the whole 10-minutes scan, with input function derived from the LV. The model parameter *K*_1_ represents perfusion, *k*_2_ represents rate of oxidative metabolism, and *V*_b_ is the fractional volume of blood in the tissue. The model is essentially the same as is applied for the analysis of ^15^O-water and ^68^Ga-DOTA. Usage of *K*_1_ from ^11^C-acetate for assessing myocardial perfusion (in addition to oxidative metabolism) has been previously validated against gold-standard ^15^O-water method in rats^10^ and in humans,^28^,^29^ and also against ^13^N-ammonia. In addition to ^11^C-acetate, it would have been possible to use ^15^O-water as a control. However, due to 2-minutes half-life of ^15^O, the transfer of ^15^O-water from the cyclotron/radiochemistry unit to the animal imaging facility was not feasible due to the long distance between sites. During this study ^13^N-ammonia was not available at our center.

In this study, we obtained the input function from the LV cavity on dynamic PET images. A direct comparison of the ^68^Ga-DOTA radioactivity concentration obtained from blood samples and PET images would be a valuable approach in a larger animal model (e.g., pigs), which would allow multiple blood samples to be taken from the same animal throughout the PET imaging. A dedicated small-animal PET/CT scanner was used to image the animals in this study, and even though it provided good resolution and we had the anatomical CT references to aid in ROI drawing, the structures being imaged were quite small (less than 10 mm in diameter). The in vivo imaging of small structures such as rat myocardium may therefore be affected by PVE, which might have influenced the in vivo quantitative PET data. When PVE is present, the apparent pixel values in PET images may be influenced by surrounding high pixel values. Other factors, such as the reconstruction algorithm and filter, scanner sensitivity, and scan duration, may also affect the accuracy of imaging data, and require evaluation.

## CONCLUSION

Our results indicate that ^68^Ga-DOTA is a potential tracer for evaluating myocardial resting perfusion in small animals. Acute MI can be detected as both reduced perfusion and increased late retention of the tracer. The myocardial kinetics of ^68^Ga-DOTA assessed from the early distribution of the chelate reflected myocardial perfusion, while delayed contrast enhancement was demonstrated at later time points, showing delayed washout in the infarcted area with an increase in the extracellular space. This approach may provide an opportunity to conduct quantitative PET perfusion imaging studies in centers distant from the cyclotron unit. Further analyses will be needed to evaluate the potential of ^68^Ga-DOTA kinetic analysis for differentiating viable and non-viable myocardial areas in a clinical setting.

## NEW KNOWLEDGE GAINED

This study demonstrates that ^68^Ga-DOTA PET has potential for in vivo quantification of MBF.

## Electronic supplementary material

Below is the link to the electronic supplementary material.

Supplementary material 1 (PPTX 2098 kb)

## Abbreviations

^68^Ga-DOTA: Gallium-68-labeled 1,4,7,10-tetraazacyclododecane-*N*′,*N*″,*N*′′′,*N*″″-tetraacetic acid
LCA: Left coronary artery
LV: Left ventricle
MBF: Myocardial blood flow
MI: Myocardial infarction
MRI: Magnetic resonance imaging
PET: Positron emission tomography
ROI: Region of interest
SUV: Standardized uptake value
1TCM: One-tissue compartmental model
