# Supplementary material for: 68Ga-DOTA chelate, a novel imaging agent for assessment of myocardial perfusion and infarction detection in a rodent model
Source: J Nucl Cardiol. 2019 May 29;27(3):891–8. doi: 10.1007/s12350-019-01752-6 (PMC7326802; doi:10.1007/s12350-019-01752-6)
Supplement: Supplementary file 1 — Supplementary material 1 (PPTX 2098 kb) [file 12350_2019_1752_MOESM1_ESM.pptx]

## Slide 1
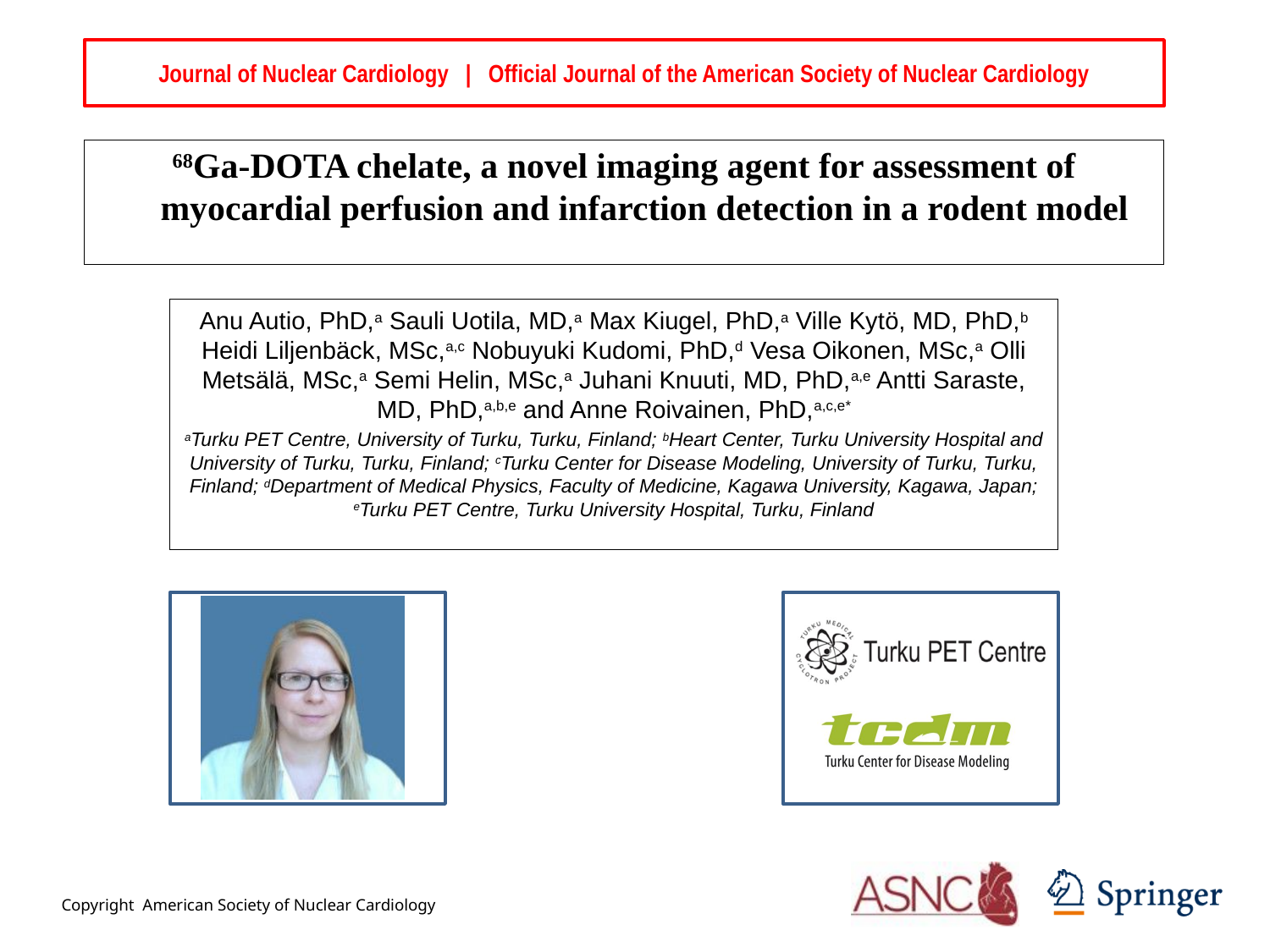

Journal of Nuclear Cardiology | Official Journal of the American Society of Nuclear Cardiology
# 68Ga-DOTA chelate, a novel imaging agent for assessment of myocardial perfusion and infarction detection in a rodent model
Anu Autio, PhD,a Sauli Uotila, MD,a Max Kiugel, PhD,a Ville Kytö, MD, PhD,b Heidi Liljenbäck, MSc,a,c Nobuyuki Kudomi, PhD,d Vesa Oikonen, MSc,a Olli Metsälä, MSc,a Semi Helin, MSc,a Juhani Knuuti, MD, PhD,a,e Antti Saraste, MD, PhD,a,b,e and Anne Roivainen, PhD,a,c,e*
aTurku PET Centre, University of Turku, Turku, Finland; bHeart Center, Turku University Hospital and University of Turku, Turku, Finland; cTurku Center for Disease Modeling, University of Turku, Turku, Finland; dDepartment of Medical Physics, Faculty of Medicine, Kagawa University, Kagawa, Japan; eTurku PET Centre, Turku University Hospital, Turku, Finland
Copyright American Society of Nuclear Cardiology

## Slide 2
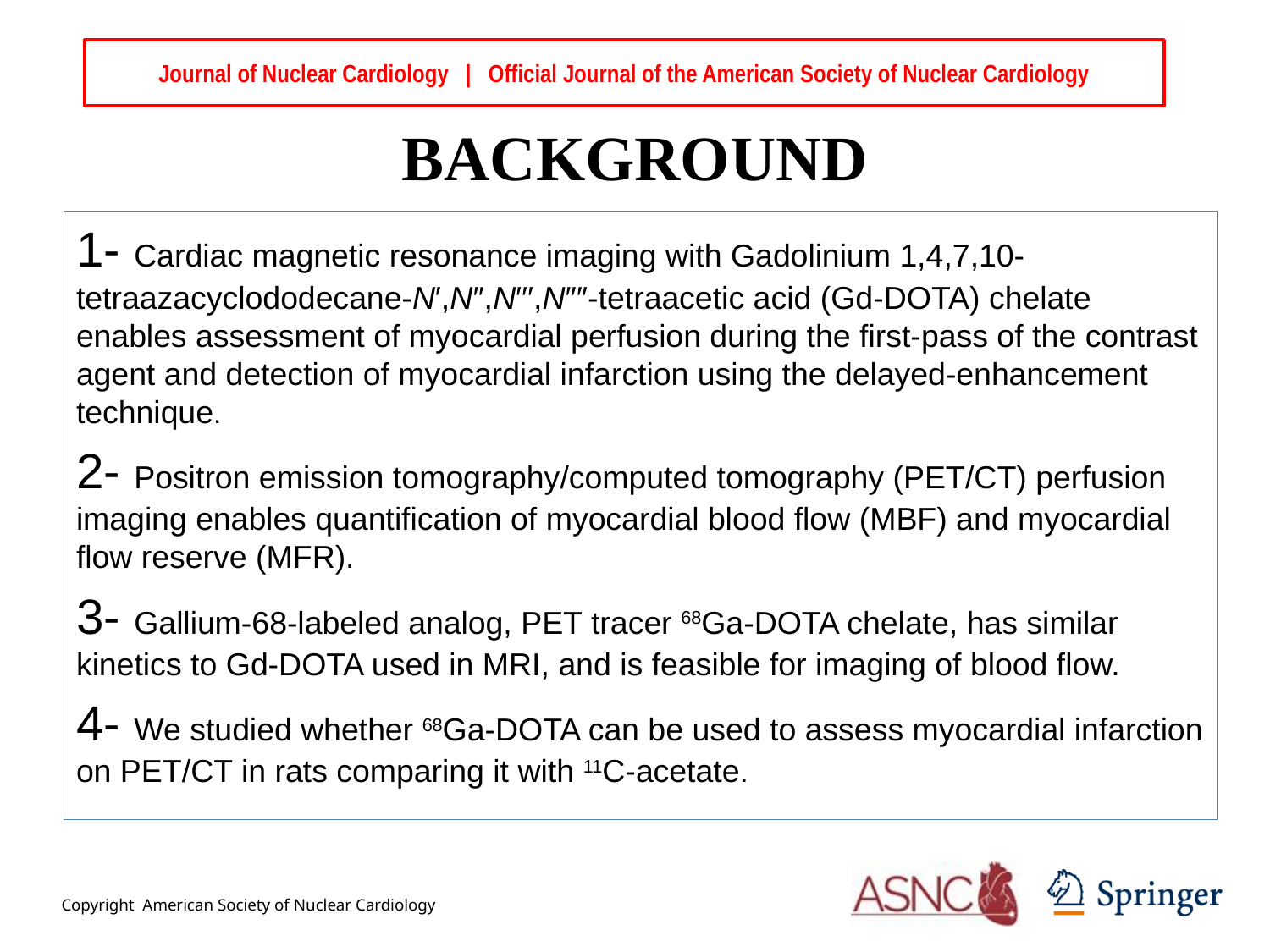

Journal of Nuclear Cardiology | Official Journal of the American Society of Nuclear Cardiology
# BACKGROUND
1- Cardiac magnetic resonance imaging with Gadolinium 1,4,7,10-tetraazacyclododecane-N′,N″,N′′′,N″″-tetraacetic acid (Gd-DOTA) chelate enables assessment of myocardial perfusion during the first-pass of the contrast agent and detection of myocardial infarction using the delayed-enhancement technique.
2- Positron emission tomography/computed tomography (PET/CT) perfusion imaging enables quantification of myocardial blood flow (MBF) and myocardial flow reserve (MFR).
3- Gallium-68-labeled analog, PET tracer 68Ga-DOTA chelate, has similar kinetics to Gd-DOTA used in MRI, and is feasible for imaging of blood flow.
4- We studied whether 68Ga-DOTA can be used to assess myocardial infarction on PET/CT in rats comparing it with 11C-acetate.
Copyright American Society of Nuclear Cardiology

## Slide 3
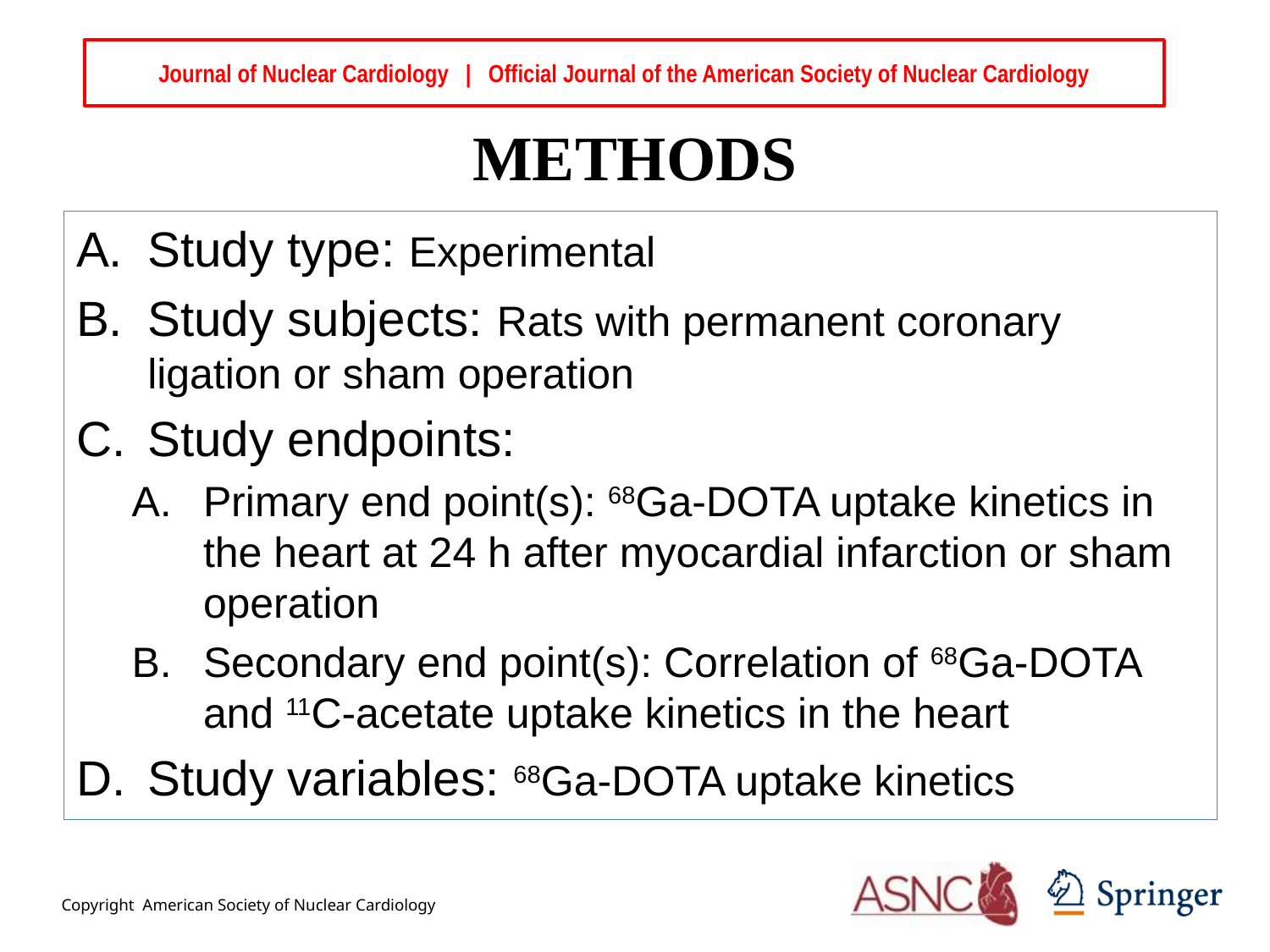

Journal of Nuclear Cardiology | Official Journal of the American Society of Nuclear Cardiology
# METHODS
Study type: Experimental
Study subjects: Rats with permanent coronary ligation or sham operation
Study endpoints:
Primary end point(s): 68Ga-DOTA uptake kinetics in the heart at 24 h after myocardial infarction or sham operation
Secondary end point(s): Correlation of 68Ga-DOTA and 11C-acetate uptake kinetics in the heart
Study variables: 68Ga-DOTA uptake kinetics
Copyright American Society of Nuclear Cardiology

## Slide 4
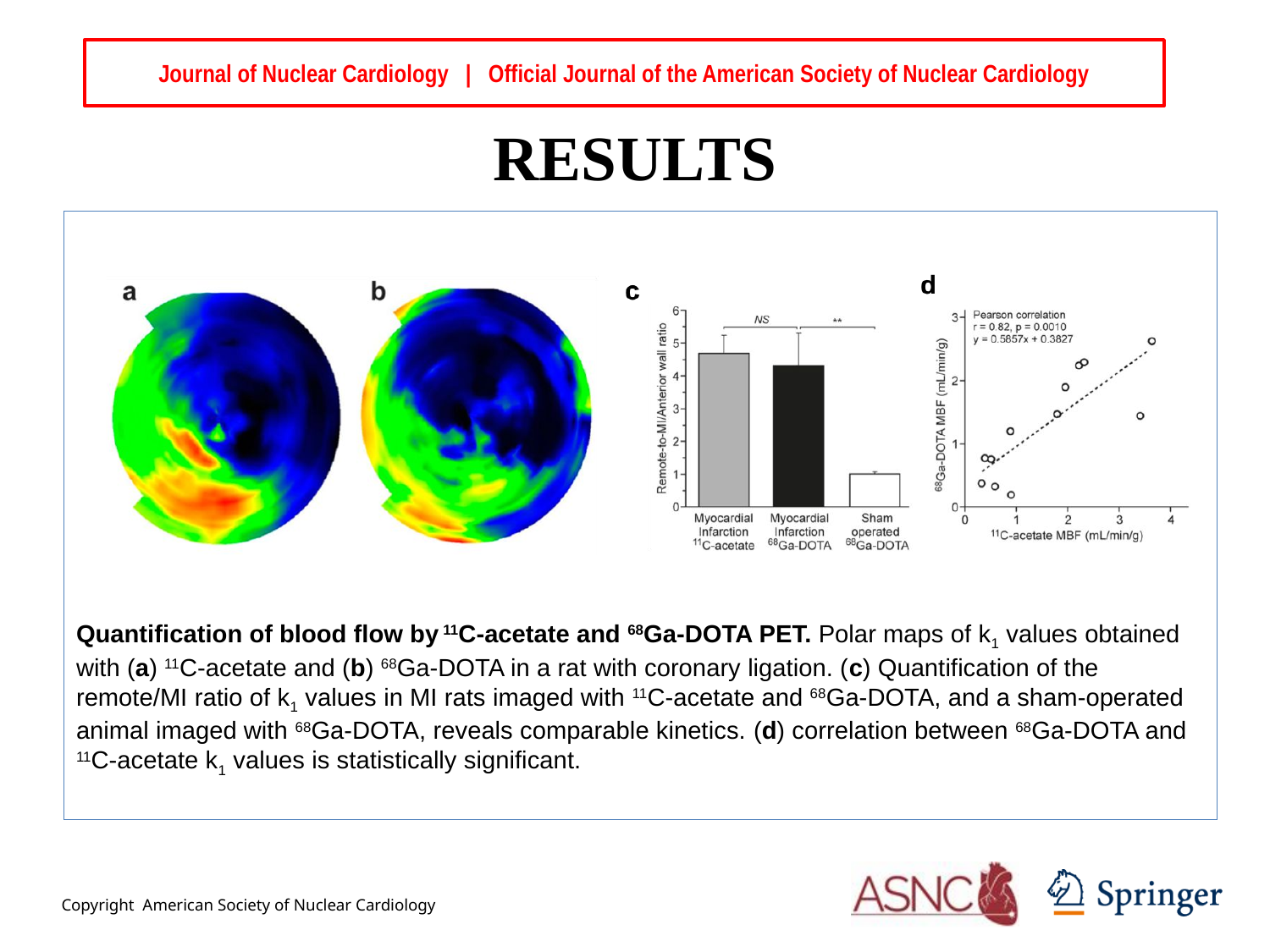

Journal of Nuclear Cardiology | Official Journal of the American Society of Nuclear Cardiology
# RESULTS
Quantification of blood flow by 11C-acetate and 68Ga-DOTA PET. Polar maps of k1 values obtained with (a) 11C-acetate and (b) 68Ga-DOTA in a rat with coronary ligation. (c) Quantification of the remote/MI ratio of k1 values in MI rats imaged with 11C-acetate and 68Ga-DOTA, and a sham-operated animal imaged with 68Ga-DOTA, reveals comparable kinetics. (d) correlation between 68Ga-DOTA and 11C-acetate k1 values is statistically significant.
Copyright American Society of Nuclear Cardiology

## Slide 5
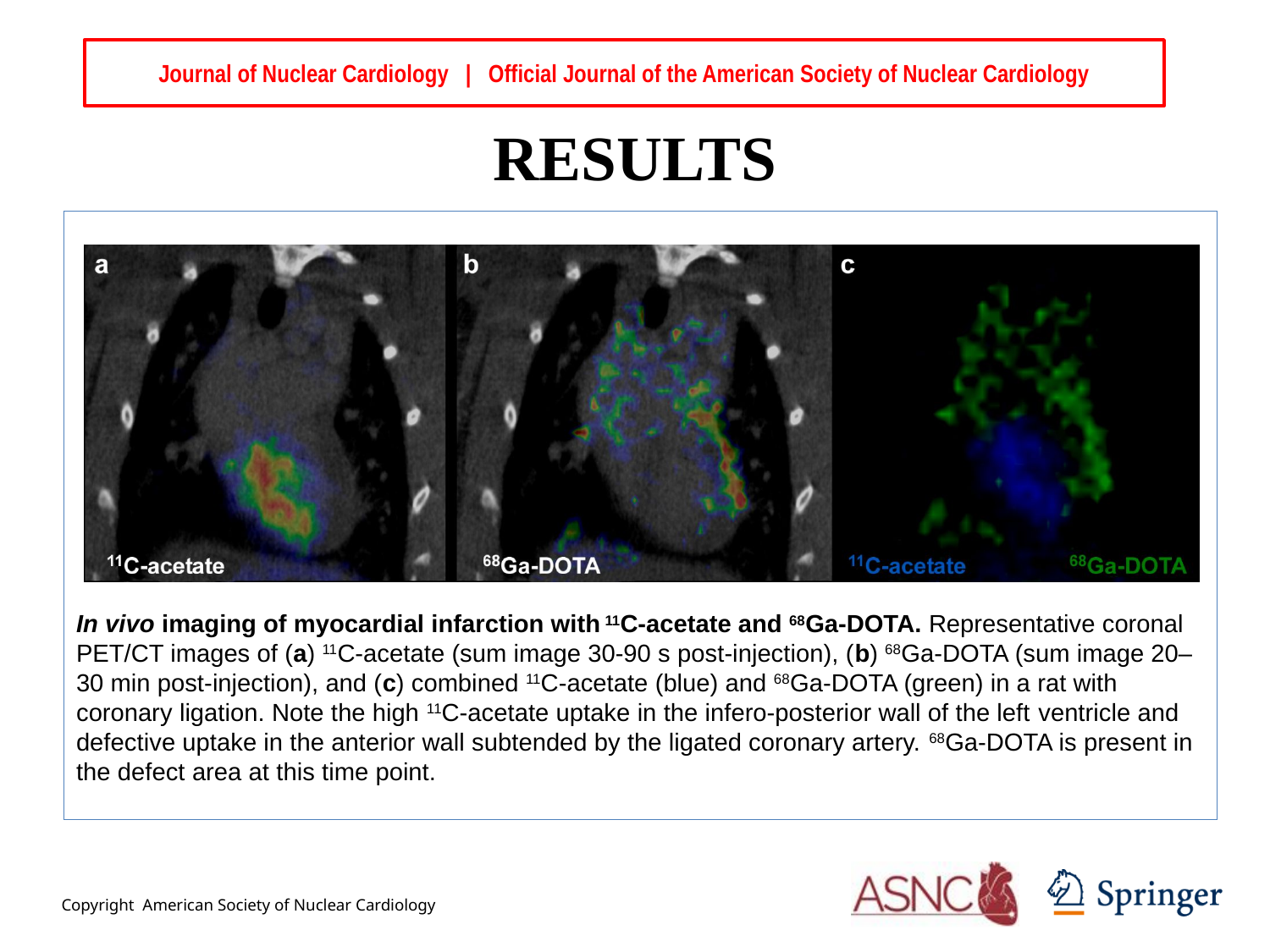

Journal of Nuclear Cardiology | Official Journal of the American Society of Nuclear Cardiology
# RESULTS
In vivo imaging of myocardial infarction with 11C-acetate and 68Ga-DOTA. Representative coronal PET/CT images of (a) 11C-acetate (sum image 30-90 s post-injection), (b) 68Ga-DOTA (sum image 20–30 min post-injection), and (c) combined 11C-acetate (blue) and 68Ga-DOTA (green) in a rat with coronary ligation. Note the high 11C-acetate uptake in the infero-posterior wall of the left ventricle and defective uptake in the anterior wall subtended by the ligated coronary artery. 68Ga-DOTA is present in the defect area at this time point.
Copyright American Society of Nuclear Cardiology

## Slide 6
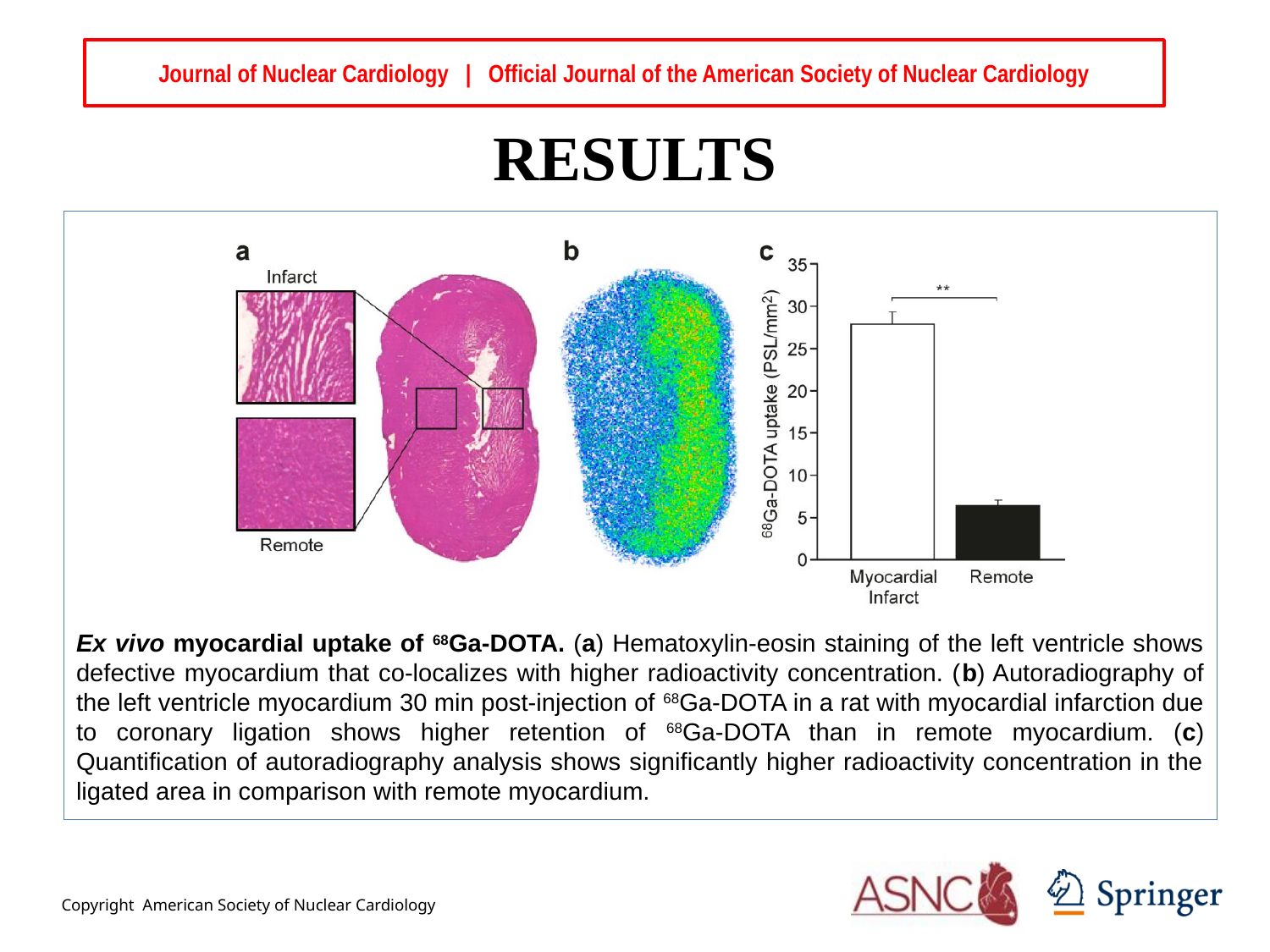

Journal of Nuclear Cardiology | Official Journal of the American Society of Nuclear Cardiology
# RESULTS
If figure, insert legen
Ex vivo myocardial uptake of 68Ga-DOTA. (a) Hematoxylin-eosin staining of the left ventricle shows defective myocardium that co-localizes with higher radioactivity concentration. (b) Autoradiography of the left ventricle myocardium 30 min post-injection of 68Ga-DOTA in a rat with myocardial infarction due to coronary ligation shows higher retention of 68Ga-DOTA than in remote myocardium. (c) Quantification of autoradiography analysis shows significantly higher radioactivity concentration in the ligated area in comparison with remote myocardium.
Copyright American Society of Nuclear Cardiology

## Slide 7
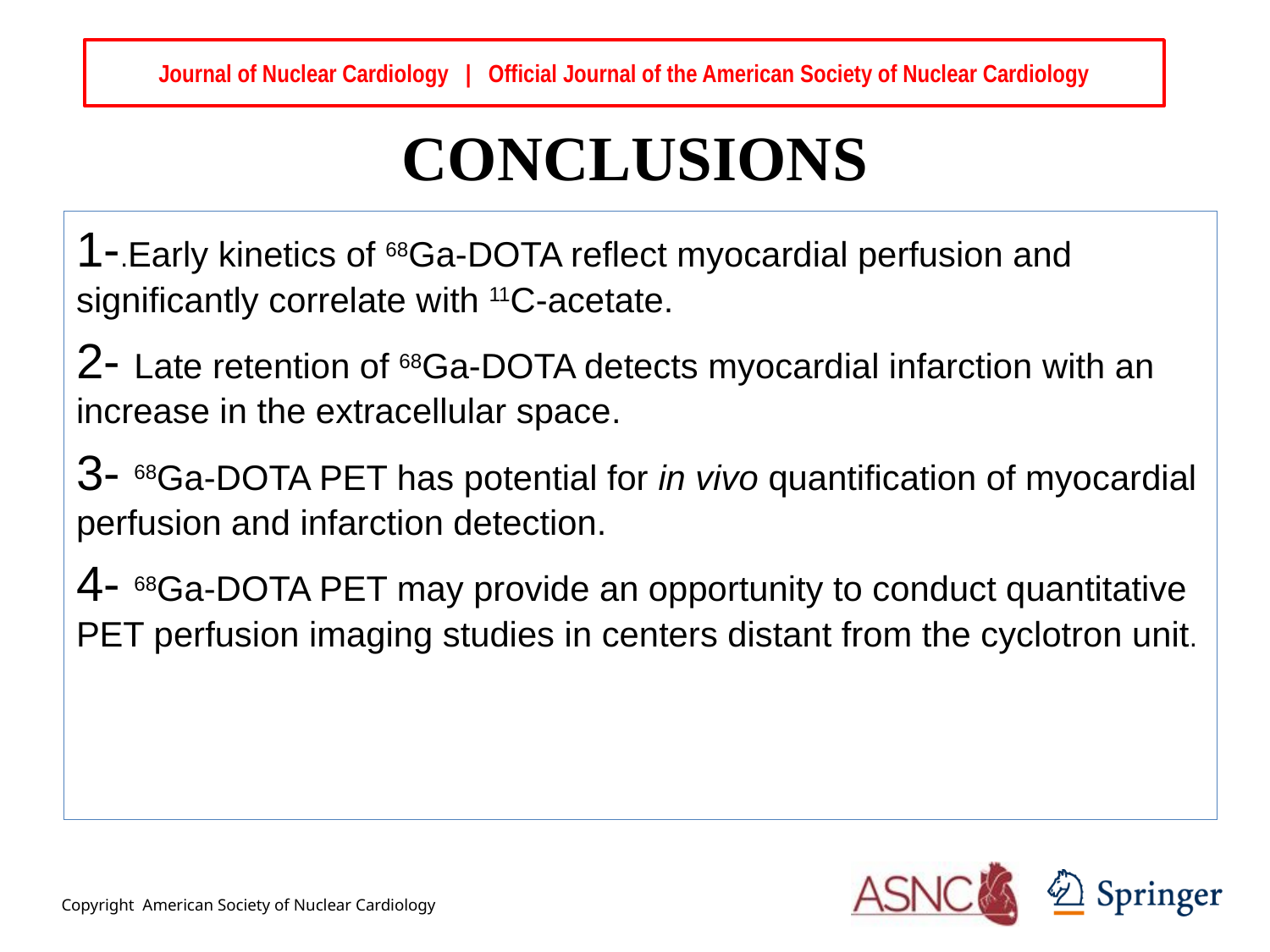

Journal of Nuclear Cardiology | Official Journal of the American Society of Nuclear Cardiology
# CONCLUSIONS
1-.Early kinetics of 68Ga-DOTA reflect myocardial perfusion and significantly correlate with 11C-acetate.
2- Late retention of 68Ga-DOTA detects myocardial infarction with an increase in the extracellular space.
3- 68Ga-DOTA PET has potential for in vivo quantification of myocardial perfusion and infarction detection.
4- 68Ga-DOTA PET may provide an opportunity to conduct quantitative PET perfusion imaging studies in centers distant from the cyclotron unit.
Copyright American Society of Nuclear Cardiology
